# CRISPR/Cas‐mediated genome editing to treat EGFR‐mutant lung cancer: a personalized molecular surgical therapy

**DOI:** 10.15252/emmm.201506006

**Published:** 2016-01-08

**Authors:** Huibin Tang, Joseph B Shrager

**Affiliations:** ^1^ Department of Cardiothoracic SurgeryDivision of Thoracic SurgeryStanford University School of MedicineStanfordCAUSA; ^2^ VA Palo Alto Healthcare SystemPalo AltoCAUSA

**Keywords:** Cancer, Genetics, Gene Therapy & Genetic Disease

## Abstract

While substantial progress has been made in the treatment of lung cancer with the development of tyrosine kinase inhibitors (TKIs) that target tumor‐driving mutations in the epidermal growth factor receptor (EGFR), nearly all patients treated with TKIs ultimately develop drug resistance due to resistance‐conferring genomic mutations. CRISPR/Cas9‐mediated genome editing is a powerful new technique that allows precise changes to be made to cells’ genomes. This technology is currently used widely in research laboratories, but it has yet to make an impact in the clinics. We suggest a potential exciting clinical application for this technical advance—allowing personalized, molecular surgery to correct or destroy the mutated *EGFR*. After detection of *EGFR* mutations in individual patients’ cancers from biopsy samples, the *EGFR*‐mutant genes will be repaired or destroyed with virus‐delivered CRISPR/Cas system. We demonstrate the feasibility of such an approach with examples from the most common primary and secondary *EGFR* mutations that are encountered. These proposed “molecular surgeries” on genomic DNA directly target the cause of the disease in a personalized and possibly permanent manner. This approach could be combined with traditional surgery, radiation therapy, or chemo/targeted therapy.

Lung cancer (LCa) is the most common type of cancer among men globally, and it is the leading cancer‐related cause of death of both men and women. In 2015, ~150,000 Americans are expected to die from this disease (American Cancer Society, 2015). Approximately 85% of lung cancers are the non‐small‐cell type (NSCLC), including adenocarcinomas and squamous cell carcinomas. Accepted LCa treatments, depending upon stage, may include surgery, radiation therapy, and/or targeted/chemotherapy. The non‐specific cytotoxicity of chemotherapy has been a long‐standing hurdle for the otherwise appealing approach of using drugs to manage cancer. However, the emergence of “targeted” drug therapy with TKIs in *EGFR*‐mutant lung adenocarcinoma has substantially mitigated this concern.

The EGFR is a membrane glycoprotein with an extracellular ligand‐binding domain, a transmembrane domain, and an intracellular tyrosine kinase domain. Ligand binding activates the intracellular tyrosine kinase, which via cascading downstream signals promotes a number of intracellular pathways that support the cancer phenotype. These include pathways underlying cellular proliferation, neovascularization, invasion and metastasis, reduced apoptosis, and activation of the Warburg effect (preferred use of aerobic glycolysis as a source of energy). Constitutive activation of the EGFR tyrosine kinase as a result of genetic mutations within it was first reported in a subgroup of lung adenocarcinoma patients (Lynch *et al*, 2004; Paez *et al*, 2004). These *EGFR* mutations are more frequent in female patients of East Asian ancestry. The most common mutations are deletions in exon 19 (del‐E746‐A750, ~50% of patients), and a point mutation in exon 21 (~40%) that substitutes leucine with arginine at codon 858 (L858R). Several drugs, such as gefitinib and erlotinib, have been developed that inhibit the tyrosine kinase activity of EGFR by competing with ATP for the ATP‐binding pocket in EGFR's tyrosine kinase domain. These drugs (TKIs) have become first‐line therapy in metastatic EGFR‐mutant NSCLC.

Although TKIs have proven to have remarkable initial efficacy in EGFR‐mutant LCa, nearly all patients unfortunately ultimately develop acquired resistance to the drugs within 2 years. This acquired drug resistance often results from a secondary mutation at position 790 in exon 20 (T790M, substituting threonine with methionine; found in ~65% of tumors with acquired resistance to TKIs). T790M‐related drug resistance may result from alteration of inhibitor binding in the ATP pocket of EGFR and restored binding affinity for ATP. To overcome drug resistance, several second‐generation drugs (afatinib/gilotrif, dacomitinib, neratinib) and third‐generation drugs (CO‐1686, AZD9291), have been developed. The second‐generation drugs are irreversible inhibitors, while the third‐generation drugs are selective to the T790M mutation. While the clinical effectiveness of these drugs has not yet been completely elucidated, preliminary data indicates that they may add about 9–13 months of progression‐free survival in appropriate patients (Cross *et al*, 2014; Politi *et al*, 2015).

Not surprisingly, we are now learning of resistance mutations (e.g., C797S) induced by third‐generation TKIs (Politi *et al*, 2015; Thress *et al*, 2015). Rather than continuing to repeat this cycle of inducing new resistance mutations via the selective pressure created by additional targeted drug therapies, the development of entirely novel approaches seems appropriate. We discuss here a new approach—personalized molecular surgery—to correct or destroy the mutated *EGFR* using CRISPR/Cas9‐mediated genome‐editing technology. CRISPR/Cas9 is an RNA‐guided gene‐editing tool that uses a bacterially derived endonuclease Cas9 (or its mutant nickase) and a single guide RNA (sgRNA) to introduce a double (or single)‐strand break at a specific location within the genome by matching the sequences between sgRNA and genomic DNA. The subsequent DNA repair then introduces an insertion or causes a deletion in the target gene through either homology‐directed repair (HDR) or non‐homologous end‐joining (NHEJ) (Cong *et al*, 2013). CRISPR/Cas‐mediated gene knockout would be expected to be more efficient than RNA interference‐mediated gene knockdown, and has until now provided a convenient laboratory tool to study gene function (Chen *et al*, 2015). More importantly, it makes it theoretically possible to repair genetic mutations in clinical diseases (Ebina *et al*, 2013; Sánchez‐Rivera & Jacks, 2015).

As proof‐of‐concept of this type of molecular surgery for lung cancer, we propose to use CRISPR/Cas9 to repair or destroy the *EGFR* gene in EGFR‐mutant NSCLC, as shown in Fig 1, with examples from the most common primary and secondary mutations. First, biopsy samples from patients will be tested for the mutations. sgRNA will then be designed (Fig 1B and C) to target the specific sequences in the mutated exons—for example, L858R in exon 21, E19del in exon 19, or the T790M resistance mutation in exon 20 (Fig 1A). To *repair* the mutated *EGFR*, we will use CRISPR/Cas9 nickase to target the DNA sequences flanking the mutation (or the whole exon if there are additional mutations in the exon). Briefly, CRISPR/Cas9 nickase creates single‐strand breaks in the genomic DNA sequence on each side of the mutations or exons (e.g., exon 19 or 21). The donor DNA harboring the wild‐type sequence of exon 19 or 21 and its right and left homologous arms will then replace the mutated sequence or exon via homologous recombination (i.e., HDR) (Fig 1B). This replacement will eradicate the carcinogenic mutations, end the constitutively activated TK activity, and thereby prevent cancer progression. This type of approach would have the greatest benefit in the primary EGFR mutations (e.g., E19del, L858R), or when there are multiple mutations in same exon (Fig 1B).

**Figure 1:**
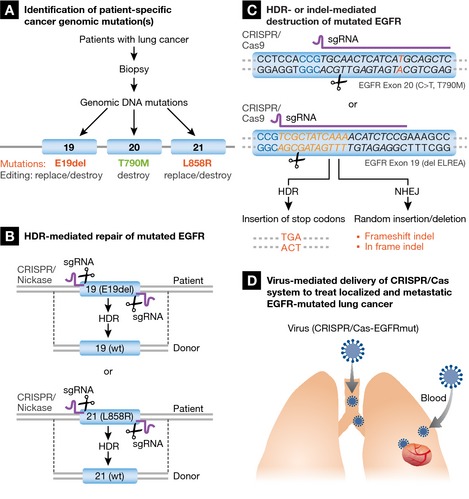
Strategy for personalized molecular surgical therapy to treat EGFR‐mutant lung cancer with CRISPR/Cas9 technology (A) Tumor tissue obtained by biopsy from a lung cancer patient. Genomic mutations in the *EGFR* gene will be identified by PCR and sequencing. The common mutations are shown, but rare mutations could be addressed as well. (B) Correction of the mutated *EGFR* gene by homology‐directed repair (HDR), substituting the mutated sequence with wild‐type sequence. Examples from exons 19 and 21 are shown. Nickase will be used to create single‐strand nicks on genomic DNA. (C) Destruction of the mutated *EGFR* gene through HDR‐ or NHEJ‐mediated truncation, insertion, and deletion. Potential sgRNA targeting sequences against exon 20 T790M (point mutation shown in red font) and exon 19 deletion (del ELREA) are shown in italics. The PAM sequence (NGG) is shown in blue font, and the deleted 15‐bp sequence formerly sat between the nucleotides labeled with yellow and black fonts. HDR‐mediated introduction of a sequence with a stop codon will yield a truncated EGFR protein lacking tyrosine kinase activity. Similarly, NHEJ would introduce a random indel leading to truncation, deletion, and/or insertion that cause destruction of tyrosine kinase activity. (D) Virus‐mediated delivery of the CRISPR/Cas9 system. CRISPR/Cas9 DNA constructs will be packaged into virus and delivered to patients via the trachea for localized cancers, or intravascularly for metastatic cancers.

To *destroy* the mutated *EGFR*, we propose to use CRISPR/Cas9 to target the mutated DNA sequence in the EGFR's tyrosine kinase domain and introduce a stop codon (HDR) or indel (NHEJ) to interrupt EGFR protein translation. The altered EGFR protein will be non‐functional and therefore lose its oncogenic activity. This mutation‐directed destruction could be applied to any mutation or deletion in the tyrosine kinase domain (from exons 18 to 24), including the more common mutations, as long as an appropriate mutation‐targeting sgRNA is available. We show in Fig 1C potential mutation‐recognition sgRNA sequences designed to target the sequences at the exon 20 T790M and the exon 19 del. CRISPR/Cas9‐mediated editing will lead to HDR‐dependent insertion of a stop codon that terminates EGFR translation at exon 19 or 20, or an NHEJ‐dependent random insertion/deletion, destroying EGFR TK activity and cancer progression.

These CRISPR/Cas9 systems (sgRNA and Cas9 expression plasmid, donor DNA plasmid) can be packaged into viruses and delivered to the patients intratracheally (for treatment of localized cancer), or intravascularly (for metastatic cancer) (Fig 1D).

These proposed “molecular surgeries” on genomic DNA in EGFR‐mutant lung cancer directly target the cause of the disease in a personalized and, it is hoped, permanent manner. A similar strategy could be employed to target other types of cancer‐driving genomic changes, such as the rearranged anaplastic lymphoma kinase (ALK) allele and K‐ras mutations. If successful, this approach would provide an alternative form of therapy that might avert the need for the costly, lengthy, and apparently endless process of developing new TKIs against new mutations. As with any therapy, this strategy could be subverted by feedback disinhibition of other cellular proliferation pathways. However, CRISPR/Cas therapy would, at a minimum, prevent the secondary genomic mutations that are the main cause of TKI resistance. Well‐designed sgRNAs, careful management of the potential off‐target effects, and efficient delivery will be necessary for the success of CRISPR/Cas‐mediated therapy. With the conceivable improvement and maturation of CRISPR/Cas technology, combining this molecular surgical approach with traditional surgery, radiation, and/or TKI treatment would have the potential to significantly improve the survival of patients with EGFR‐mutant NSCLC.
